# New digital tool to facilitate subcutaneous insulin therapy orders: an inpatient insulin dose calculator

**DOI:** 10.1186/s13098-015-0111-7

**Published:** 2015-12-21

**Authors:** Marcos Tadashi Kakitani Toyoshima, Alexandre Barbosa Câmara de Souza, Sharon Nina Admoni, Priscilla Cukier, Simão Augusto Lottenberg, Ana Claudia Latronico, Márcia Nery

**Affiliations:** Serviço de Endocrinologia e Metabologia do Hospital das Clínicas da Faculdade de Medicina da, Universidade de São Paulo, Av. Dr. Enéas Carvalho de Aguiar, 255-7º andar, Sala 7037, São Paulo, SP CEP: 05403-900 Brazil; Unidade de Oncologia Endócrina do Instituto do Câncer do Estado de São Paulo-Hospital das Clínicas da Faculdade de Medicina da, Universidade de São Paulo, Av. Dr. Arnaldo, 251-5º andar, São Paulo, SP CEP: 01255-000 Brazil; Centro de Medicina Preventiva, Hospital Israelita Albert Einstein, Av. Brasil, 953, São Paulo, SP CEP: 01431-000 Brazil

**Keywords:** Diabetes mellitus, Hyperglycemia, Hospitalization, Insulin

## Abstract

**Background:**

Inpatient hyperglycemia is associated with adverse outcomes in hospitalized patients, with or without known diabetes. The adherence to American College of Endocrinology and American Diabetes Association guidelines recommendations for inpatient glycemic control is still poor, probably because of their complexity and fear of hypoglycemia.

**Objective:**

To create software system that can assist health care providers and hospitalists to manage the insulin therapy orders and turn them into a less complicated issue.

**Methods:**

A software system was idealized and developed, according to recommendations of major consensus and medical literature.

**Results:**

HTML software was developed to be readily accessed from a workstation, tablet or smartphone. Standard initial daily total dose of insulin was 0.4 units/kg and could be modified by distinct factors, such as chronological age, renal and liver function, and high dose corticosteroids use. Insulin therapy consisted of basal, prandial and correction insulin according to nutritional support, glycemic control and outpatient treatment for diabetes. Human insulin or insulin analogues could be options for insulin therapy. Sensitivity factor was based on 1800 Rule for rapid-acting insulin and the 1500 Rule for short-acting insulin. Insulin-naïve patients with initial BG level less than 250 mg/dL were considered to have an initial step-wise approach with prandial and correction insulin. The calculator system has allowed insulin dose readjustments periodically, according to daily average blood glucose measurements.

**Conclusion:**

We developed software that can be a useful tool for all public hospitals, where generally human insulin is the only available.

## Brief report

Inpatient hyperglycemia is associated with adverse clinical outcomes in hospitalized patients, with or without known previous diabetes mellitus (DM), including prolonged hospital stay, infection, disability after hospital discharge, death and higher health care costs [1–4]. Even in patients without DM, hyperglycemia may be an acute manifestation of critical and surgical illness that results from the metabolic and hormonal changes, in response to injury and stress [2].

The American College of Endocrinology (ACE) and the American Diabetes Association (ADA) Task Force on Inpatient Glycemic Control recommend fasting blood glucose (BG) level less than 140 mg/dL (7.8 mmol/L) and a random BG level less than 180 mg/dL (10.0 mmol/L), without excess hypoglycemia, for the majority of non-critically ill patients treated with insulin. In order to avoid hypoglycemia, readjustments in the insulin regimen were considered if BG levels decline below 100 mg/dL (5.6 mmol/L). It was suggested for BG testing: before meals and at bedtime in patients who were eating, or every 4–6 h in patients who were receiving nothing by mouth (nil per os—nPO) or receiving continuous enteral feeding [4]. Despite the fact that many studies have shown solid evidence in support of inpatient glycemic control, BG control remains deficient and overlooked [2].

Subcutaneous insulin is the preferred therapeutic agent for BG control in hospitalized patients in non-critical care setting [2, 5, 6]. The sliding scale insulin (SSI) is the most commonly chosen insulin therapy regimen. However, this regimen results in great glycemic variability with undesirable levels of hypoglycemia and hyperglycemia [5]. Clinical practice guidelines have recommended the use of insulin regimen with combined basal and short or rapid-acting insulin (basal bolus approach) as the preferred insulin regimen for the management of hyperglycemia in hospitalized patients in non-critical care setting. Basal bolus insulin therapy based on BG results is safe and efficacious in the management of inpatient hyperglycemia [11].

Despite the benefits of a basal bolus regimen in improving glycemic control in non-critically ill patients, many health care providers are still reluctant to integrate this approach into their clinical practice, probably because of its complexity and a fear of hypoglycemia. Moreover, the management of diabetes in the hospital is generally considered less important compared with the condition that prompted admission and clinical inertia rules in glycemia management in the hospital [7–9]. In Brazil, the inpatient BG management is also deficient, whereas DM is the fifth most common reason for hospitalizations and ranked among the 10 major causes of mortality [3]. Recently, we analyzed 368 hospitalized patients at Hospital das Clínicas da Faculdade de Medicina da Universidade de São Paulo and 23 % of patients had previous diagnosis of diabetes mellitus, but only 1 % of them had received basal-bolus as insulin therapy regimen [10].

There is still a lack of clinical studies on the use of neutral protamine Hagedorn (NPH) and regular insulin (available in public health care system in Brazil) for inpatient treatment of hyperglycemia. Most of prospective randomized studies used insulin analogues as insulin therapy for hospitalized patients. Randomized studies showed that a basal bolus regimen with insulin analogues resulted in equivalent glycemic control and frequency of hypoglycemia compared to treatment with human insulin. The number of episodes of hypoglycemia less than 40 mg/dL was low in both groups, but not statistically significant higher in the group with NPH and regular insulin [11, 12].

**Fig. 1:**
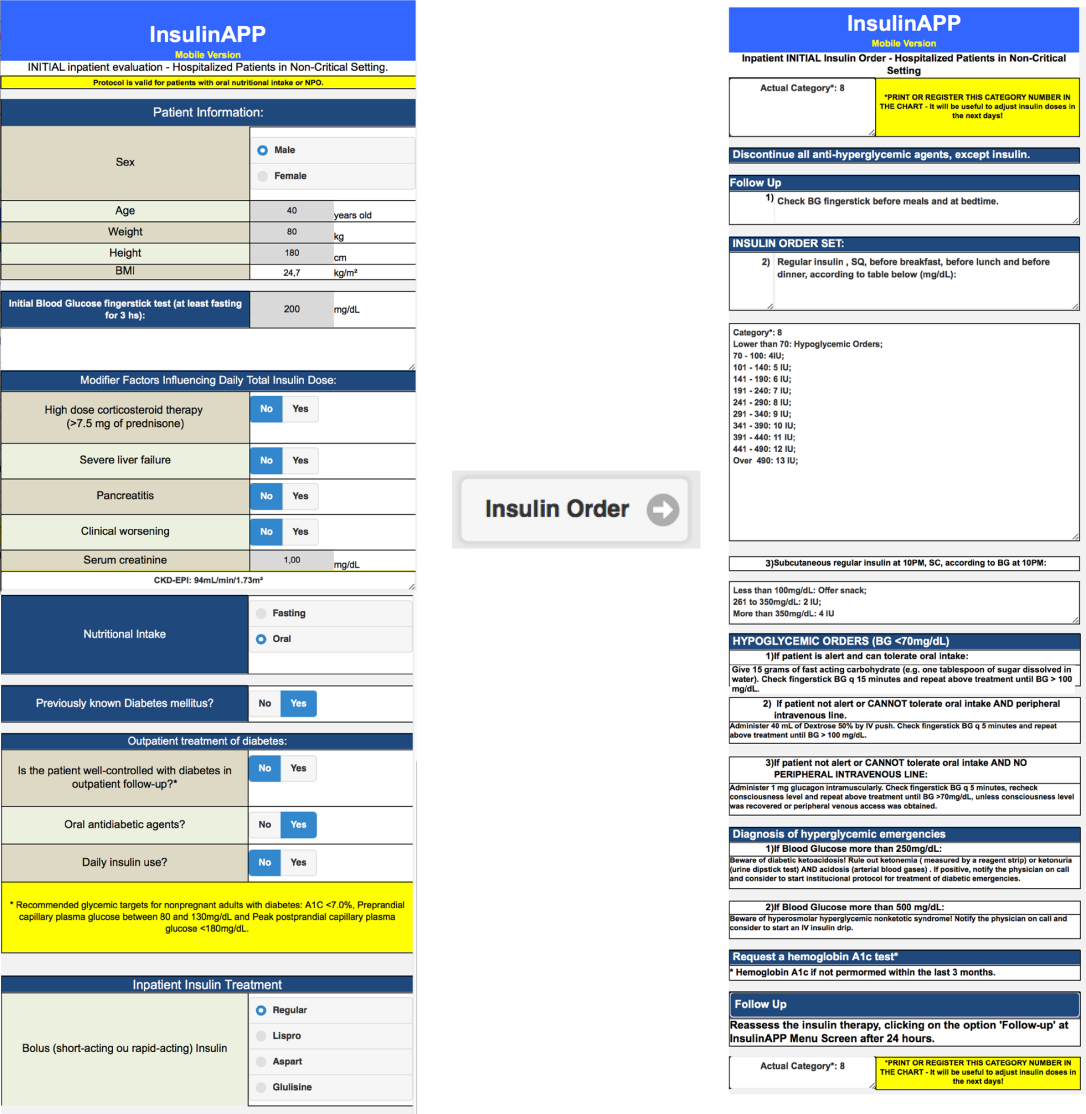
Screenshot of the application showing an example of initial management of patient with hyperglycemia and the respective prescription

**Fig. 2:**
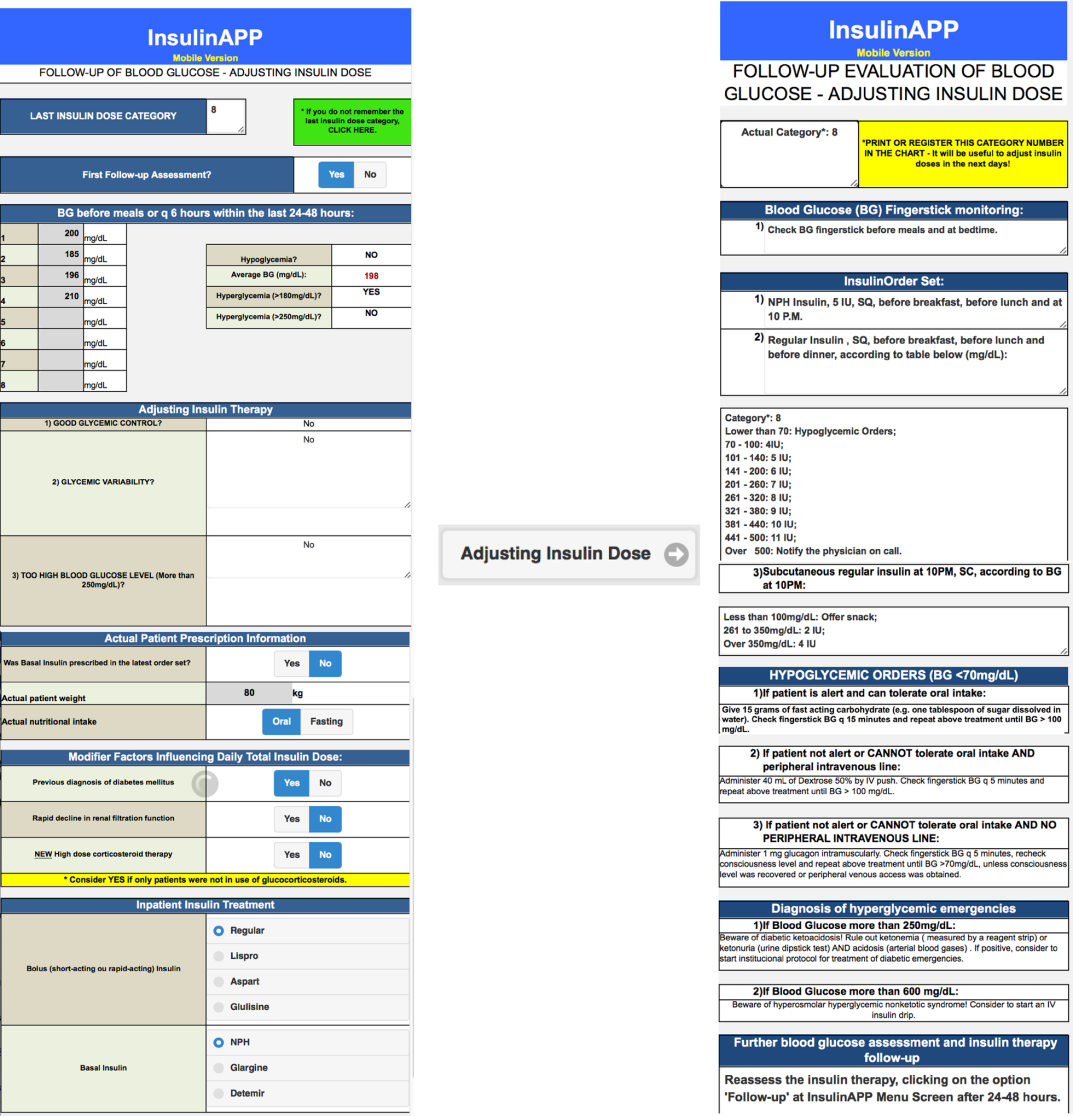
Screenshot of the application showing an example of follow-up of blood glucose measurements and the respective prescription with adjust of insulin regimen or dose

The relationship between hyperglycemia and hospital outcomes is well-established [8] and major consensus recommends that insulin therapy should be prescribed for all patients with hyperglycemia, preferentially with basal-bolus regimen. Accordingly, we previously implemented a paper-based glycemic control protocol in hospitalized patients that works with flowcharts and tables, the same way as most of hospitals has done. Because of the adherence had been still low, we idealized a software program that could assist the health care providers and hospitalists to manage the insulin therapy orders in the hospital setting and turn them into a less complicated issue. The software was developed as an HTML application that can be readily accessed through a network using a workstation, tablet or smartphone. It could be run on Windows PC, Macintosh, iOS or Android devices (Figs. 1, 2).

In this new software system, glycemic targets were defined according to ACE/ADA guidelines. Average BG between 100 and 140 mg/dL was defined as target for the non-critically ill-patients BG control.

The category dose insulin was defined to become easier to remind the total daily dose of insulin (TDDI). The standard TDDI was 0.4 units/kg and was defined as insulin dose category number ´8´. One category could be increased or decreased by 0.05 units/kg, according to modifier factors described ahead. A lower category dose was considered for patients at risk of hypoglycemia, such as elderly patients or those with renal or liver impairment. Higher dose was considered for patients with higher insulin resistance, such as obese patients or those in use of high dose of corticosteroids [9, 13]. Every modifier factor could affect the insulin dose category independently and the final category was defined according to their combination. BG testing was recommended before meals and at bedtime in patients who were eating, or every 6 h in patients who were receiving nothing by mouth (nil per os—NPO) or receiving continuous enteral feeding (Fig. 1).

**Fig. 3:**
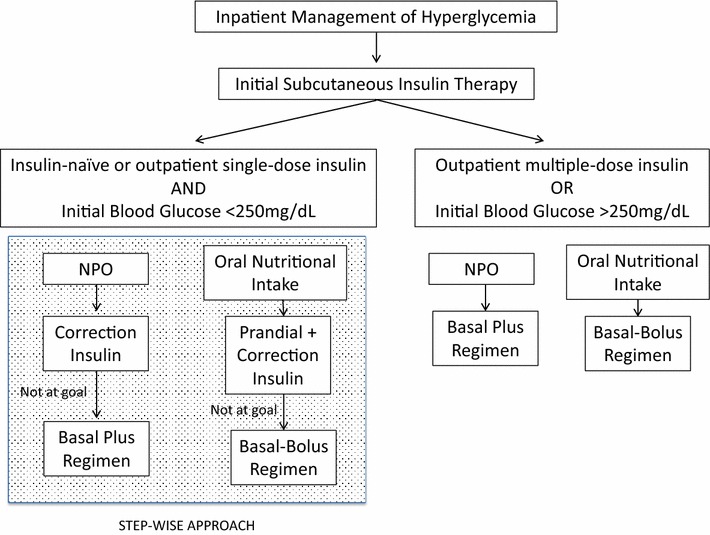
Initial insulin therapy for hyperglycemia in hospitalized patients. Insulin therapy regimen was chosen according to initial blood glucose, outpatient diabetes treatment and nutritional support. A step-wise approach was considered for patients who were insulin-naïve, in use of outpatient single-dose insulin treatment and with initial BG level less than 260mg/dL

Scheduled subcutaneous insulin therapy consisted of the following components: basal insulin administered as NPH insulin twice or three times a day or glargine and detemir insulin once a day; and prandial and correction insulin administered as short-acting (regular) or as rapid-acting insulin (lispro, aspart, glulisine) prior to major meals (breakfast, lunch and dinner) or four times a day (every 6 h), if NPO or with enteral feeding. Hyperglycemic or DM patients who were eating received about 50 % of the TDDI as basal insulin and 50 % as prandial (or nutritional) insulin in divided doses administered with major meals (basal-bolus regimen). Correction dose was combined with prandial insulin dose to compensate for hyperglycemia before meals. Patients who were not eating (at NPO status) were considered to receive basal (50 % of the TDDI) and correction-dose insulin, as well called basal plus regimen [14]. The prandial insulin dose was withdrawn. Patients who were insulin-naïve or in use of outpatient insulin treatment with total daily dose less than 0.2 IU/kg, and initial BG level less than 250 mg/dL (14.4 mmol/L), an initial step-wise approach was considered with prandial insulin and correction dose (Fig. 3). After revaluation in 24–48 h, basal insulin was included if average BG has been more than 180 mg/dL [15]. To calculate the sensitivity (or correction or supplemental) factor and consequently correction insulin-dose, the 1800 Rule for rapid-acting insulin, and the 1500 Rule for short-acting insulin were used [16].

Patients that were eating and had hyperglycemia at bedtime, supplemental rapid or short-acting insulin was recommended as follow: two units for BG 250–350 mg/dL (13.9–19.4 mmol/L) and four units for BG more than 350 mg/dL (more than 19.4 mmol/L). A randomized trial of insulin supplementation for correction of inpatient bedtime hyperglycemia didn’t show that bedtime insulin dose to correct mild and moderate hyperglycemia (less than 350 mg/dL) in patients under basal-bolus regimen was not associated with significant improvement in glycemic control or frequency of hypoglycemia episodes [17].

Some safety alerts were associated with the program to help to recognize diabetes emergencies. It has been available specific orders for hypoglycemia episodes, including glucagon use if patient was not alert and with no peripheral intravenous line. If any blood glucose measurement has been more than 250 mg/dL, a message to rule out diabetes ketoacidosis has been shown up. Insulin dose readjustments were recommended to be done within 24–48 h (Fig. 2). If average BG has been more than 140 mg/dL, the insulin dose category was increased. In case of average BG measurements less than 100 mg/dL, the software has recommended to reduce the insulin dose category. For patients with great glycemic variability, defined as hypoglycemia and hyperglycemia episodes in a short period of time, the software has recommended to consider Endocrine or Diabetes Team Consultation to individualize the treatment.

In conclusion, we developed the first Brazilian application software that can use human insulin (NPH and regular) or insulin analogues (glargine, detemir, lispro, aspart and glulisine) as options for inpatient hyperglycemia management, and therefore it can be a useful tool for all public hospitals. We hope it can assist the health care providers and hospitalists to manage the insulin therapy orders in the hospital setting, and turn them into a less complicated issue, with improved adherence to guidelines recommendations. The software did not replace the physicians and their decisions, but could act to extend their knowledge and to help with the insulin dose prescription. Further studies are still necessary to assess this electronic-based insulin protocol adherence.

## Abbreviations

ACE: American College of Endocrinology
ADA: American Diabetes Association
BG: blood glucose
DM: diabetes mellitus
ICU: intensive care unit
NPH: neutral protamine Hagedorn
NPO: nothing by mouth
TDDI: total daily dose of insulin
